# Editorial: Endocrine Modulators of Neurological Processes: Potential Treatment Targets of Pediatric Neurological Diseases

**DOI:** 10.3389/fendo.2021.655290

**Published:** 2021-02-18

**Authors:** Hong Ni, Giuseppe Biagini, Dinesh Upadhya, Alessandro Capuano

**Affiliations:** ^1^ Division of Brain Science, Institute of Pediatric Research, Children’s Hospital of Soochow University, Suzhou, China; ^2^ Department of Biomedical, Metabolic and Neural Sciences, University of Modena and Reggio Emilia, Modena, Italy; ^3^ Centre for Molecular Neurosciences, Kasturba Medical College, Manipal Academy of Higher Education, Manipal, India; ^4^ Movement Disorders Clinic, Neurology Unit, Department of Neuroscience, Bambino Gesù Children's Hospital (IRCCS), Rome, Italy

**Keywords:** biomarker, endocrine-modulating therapy, ghrelin, ketogenic diet, leptin, melatonin, pediatric neurology

## Introduction

Over the last decade, significant progress has been made in understanding the effects of neuroendocrine regulators, represented by brain gut peptides, leptin, melatonin, as well as ketogenic diet (KD), on brain development, brain injury repair and early warning. This Research Topic contains 10 articles, contributed by 40 authors, from 17 countries (plus 28 experts participating in the review and editing), focusing on most recent understanding of the effects of neuroendocrine regulators on pediatric neurological diseases, including the predictive value of ghrelin, leptin, and other adipokines in the early diagnosis and intervention on pediatric neuropathy, the progress of basic research and the potential translational medicine value, etc.

Gastrointestinal peptides play important roles by regulating feeding and energy homeostasis. Ghrelin is a multifaceted gut hormone that is famously known as a “hunger hormone,” just the opposite of cholecystokinin (CCK), which is referred to as a “satiety factor.” Both hormones act *via* the peripheral vagal afferent and interact to modulate feeding regulation (1). The anticonvulsant effects of ghrelin and CCK have been reported (2, 3). Notably, ghrelin is expected to be an effective therapy for lean patients with cachexia caused by chronic heart failure, anorexia nervosa, and functional dyspepsia (4). In this Research Topic, Marchiò et al. evaluated ghrelin and growth during 1 year of ketogenic diet (KD). They examined a small cohort of six children (two males and four females, age range 3–10.4 years) affected by refractory epilepsy, who received the KD as add-on treatment. The results showed that ghrelin plasma levels are consistently reduced in children with refractory epilepsy and maintained on the KD. This change was associated with low growth indexes in the majority of patients.

Although the mechanism of KD’s anti-epileptogenic and neuroprotective effects has been studied in recent years (5, 6), there is still a lack of spectral molecular expression research. Here a proteomics study by Zheng et al. analyzed the effects of KD against lithium chloride/pilocarpine-induced status epilepticus (SE) in rats. Seventy-nine proteins in hippocampus showing a significant change in abundance between SE and control (Ctr) groups were reciprocally regulated in the SE + KD group compared to the SE group (i.e., the seizure-induced change was reversed by KD). Of these, five (dystrobrevin, centromere protein V, oxysterol-binding protein, tetraspanin-2, and progesterone receptor membrane component 2) were verified by parallel reaction monitoring. Kyoto Encyclopedia of Genes and Genomes (KEGG) pathway analysis indicated that proteins of the synaptic vesicle cycle pathway were enriched both among proteins differing in abundance between SE and Ctr groups as well as between SE + KD and SE groups.

Chen et al. investigated the effects of different zinc concentrations in the diet on long-term neurobehavioral and seizure thresholds following lithium chloride/pilocarpine-induced developmental seizures. They found that zinc supplementation for 4 weeks significantly improved injury-related changes, and rescued abnormalities in GPR39, ZnT-3, and MBP expression in the hippocampus, which suggests that the role of zinc ions in developmental convulsive brain injury may not be purely excitotoxic as previously thought (7–9), but may play a protective role in the brain as mentioned earlier (10). In a perspective article by Doenyas, a more comprehensive approach termed the “gut-immune-endocrine-brain” axis, is taken, based on which a personalized treatment plan for autism spectrum disorder (ASD) is presented.

Another clinical Cross-Sectional Study by Chen et al. in the present Research Topic investigated the association among plasma adipokines, mainly leptin, visfatin, adiponectin, or IL-6, and the prognosis of febrile seizures (FS). The main findings were that serum adiponectin and IL-6 levels were significantly higher in the FS group than in the FC and HC groups, while there was no statistical difference between the FC and HC groups, indicating that higher plasma levels of IL-6 and adiponectin could serve as an additional biomarker in the early treatment or follow-up of FS children.

Melatonin is an indoleamine secreted by the pineal gland. It can be used for the treatment of children with developmental disorders, such as ASD and attention deficit/hyperactivity disorder to improve their sleep disturbance (11, 12). Animal experiments and limited human data have confirmed the neuroprotective effect of melatonin on hypoxic-ischemic (HI) or developmental seizure-induced excitotoxic brain damage (13–15). Here, two studies have explored the molecular signaling mechanism of the neuroprotective effect of melatonin through the *in vitro* glutamate excitotoxic injury model and the *in vivo* cerebral palsy model. Wang et al. investigated the impact of melatonin on the parameters of glutamate cytotoxicity in mouse HT22 hippocampal neurons and tested the hypothesis that melatonin confers neuroprotective effects *via* mitochondrial oxidative stress/autophagy signaling. The findings indicate that melatonin exerts neuroprotective effects against glutamate-induced excitotoxicity by reducing mitophagy-related oxidative stress and maintaining mitochondrial function. Sun et al. used plppr5 knockout (plppr5-/-) mice and their wild-type littermates to establish a model of HI injury to further explore the effects of melatonin on brain injury and the role of plppr5 in this treatment in an HI model, which mainly focuses on cognition, exercise, learning and memory. They found that plppr5 knockout aggravated HI damage and partially weakened the neuroprotective effect of melatonin in some aspects (such as novel object recognition tests and partial nerve reflexes).

Interactions between the brain and distinct adipose depots have a key role in maintaining energy balance, thereby promoting brain development, among them, leptin play a key role (16). Recently, there has been renewed interest in the role of leptin in repairing developmental brain damage (17, 18). Here, a review article by Fujita and Yamashita summarize novel functions of leptin in animal models of neurodegenerative diseases. Specifically, they focus on the emerging evidence for the role of leptin in non-neuronal cells in the central nervous system, including astrocytes, microglia, and oligodendrocytes, which provides helpful information to establish therapeutic strategies to address the neurological diseases.

In addition, the study by Zhao et al. explored the significance of the α2 isoform of Na+/K+-ATPase in the regulation of the electrophysiological properties of skeletal muscle cells by β-Catenin. The review article by Wu et al. discussed the emerging roles of long non-coding RNAs in chronic neuropathic pain.

In conclusion, the present clinical and basic studies allow us a better understanding of the effects of endocrine modulators on pediatric neurological diseases. We hope that the information gathered from this Research Topic will help promote clinical translational medical research to better prevent and treat these injuries in the near future.

## Author Contributions

HN wrote the draft. GB, DU, and AC reviewed the manuscript. All authors contributed to the article and approved the submitted version.

## Funding

This work was supported by the National Natural Science Foundation of China (81871024 and 81471337) and the key talent’s subsidy project in science and education of Department of Public Health of Jiangsu Province (ZDRCC2016008).

## Conflict of Interest

The authors declare that the research was conducted in the absence of any commercial or financial relationships that could be construed as a potential conflict of interest.

## References

[1] Date Y Toshinai K Koda S Miyazato M Shimbara T Tsuruta T. Peripheral interaction of ghrelin with cholecystokinin on feeding regulation. Endocrinology (2005) 146:3518–25. 10.1210/en.2004-1240 15890776

[2] Lee SY Soltesz I. Cholecystokinin: a multi-functional molecular switch of neuronal circuits. Dev Neurobiol (2011) 71:83–91. 10.1002/dneu.20815 21154912 PMC3005241

[3] Coppens J Aourz N Walrave L Fehrentz JA Martinez J De Bundel D. Anticonvulsant effect of a ghrelin receptor agonist in 6Hz corneally kindled mice. Epilepsia (2016) 57:e195–9. 10.1111/epi.13463 27378373

[4] Ueno H Shiiya T Nakazato M. Translational research of ghrelin. Ann N Y Acad Sci (2010) 1200:120–7. 10.1111/j.1749-6632.2010.05509.x 20633140

[5] Tian T Li LL Zhang SQ Ni H. Long-Term Effects of Ketogenic Diet on Subsequent Seizure-Induced Brain Injury During Early Adulthood: Relationship of Seizure Thresholds to Zinc Transporter-Related Gene Expressions. Biol Trace Elem Res (2016) 174:369–76. 10.1007/s12011-016-0730-3 27147436

[6] Ni H Jiang YW Tao LY Jin MF Wu XR. ZnT-1, ZnT-3, CaMK II, PRG-1 expressions in .hippocampus following neonatal seizure-induced cognitive deficit in rats. Toxicol Lett (2009) 184:145–50. 10.1016/j.toxlet.2008.11.003 19059322

[7] Ni H Jiang YW Tao LY Cen JN Wu XR. Effects of penicillin-induced developmental epilepticus on hippocampal regenerative sprouting, related gene expression and cognitive deficits in rats. Toxicol Lett (2009) 188:166–6. 10.1016/j.toxlet.2009.04.002 19446251

[8] Ni H Feng X Gong Y Tao LY Wu XR. Acute phase expression pattern of ZnTs, LC3, and beclin-1 in rat Hippocampus and its regulation by 3-methyladenine following recurrent neonatal seizures. Biol Trace Elem Res (2011) 143:320–31. 10.1007/s12011-010-8836-5 20838925

[9] Ni H Li C Feng X Cen JN. Effects of forced running exercise on cognitive function and its relation to zinc homeostasis-related gene expression in rat hippocampus. Biol Trace Elem Res (2011) 142:704–12. 10.1007/s12011-010-8793-z 20703826

[10] Bruni O Alonso-Alconada D Besag F Biran V Braam W Cortese S. Current role of melatonin in pediatric neurology: clinical recommendations. Eur J Paediatr Neurol (2015) 19:122–33. 10.1016/j.ejpn.2014.12.007 25553845

[11] Maras A Schroder CM Malow BA Findling RL Breddy J Nir T. Long-Term Efficacy and Safety of Pediatric Prolonged-Release Melatonin for Insomnia in Children with Autism Spectrum Disorder. J Child Adolesc Psychopharmacol (2018) 28:699–710. 10.1089/cap.2018.0020 30132686 PMC6306655

[12] Paprocka J Kijonka M Rzepka B Sokół M. Melatonin in Hypoxic-Ischemic Brain Injury in Term and Preterm Babies. Int J Endocrinol (2019) 2019:9626715. 10.1155/2019/9626715 30915118 PMC6402213

[13] Barghout MS Al-Shahawy AK El Amrousy DM Darwish AH. Comparison Between Efficacy of Melatonin and Diazepam for Prevention of Recurrent Simple Febrile Seizures: A Randomized Clinical Trial. Pediatr Neurol (2019) 101:33–8. 10.1016/j.pediatrneurol.2019.01.010 31521449

[14] Ni H Sun Q Tian T Feng X Sun BL. Prophylactic treatment with melatonin before recurrent neonatal seizures: effects on long-term neurobehavioral changes and the underlying expression of metabolism-related genes in rat hippocampus and cerebral cortex. Pharmacol Biochem Behav (2015) 133:25–30. 10.1016/j.pbb.2015.03.012 25818576

[15] Caron A Lee S Elmquist JK Gautron L. Leptin and brain-adipose crosstalks. Nat Rev Neurosci (2018) 19:153–65. 10.1038/nrn.2018.7 PMC5962962 29449715

[16] Jin MF Ni H Li LL. Leptin maintained zinc homeostasis against glutamate- induced excitotoxicity by preventing mitophagy-mediated mitochondrial activation in HT22 hippocampal neuronal cells. Front Neurol (2018) 9:322:322. 10.3389/fneur.2018.00322 29867731 PMC5954240

[17] Ni H Chen SH Li LL Jin MF. Leptin treatment prevents long-term abnormalities in cognition, seizure threshold, hippocampal mossy fiber sprouting and ZnT3/CB-D28k expression in a rat developmental “twist” seizure model. Epilepsy Res (2018) 139C:164–70. 10.1016/j.eplepsyres.2017.12.009 29287786

[18] Ni H Zhao DJ Tian T. Ketogenic diet change cPLA2/clusterin and autophagy related gene expression and correlate with cognitive deficits and hippocampal MFs sprouting following neonatal seizures. Epilepsy Res (2016) 120:13–8. 10.1016/j.eplepsyres.2015.11.021 26709877

